# Ideal cardiovascular health predicts lower risk of abnormal liver enzymes levels in the Chilean National Health Survey (2009–2010)

**DOI:** 10.1371/journal.pone.0185908

**Published:** 2017-10-19

**Authors:** Antonio García-Hermoso, Anthony C. Hackney, Robinson Ramírez-Vélez

**Affiliations:** 1 Laboratorio de Ciencias de la Actividad Física, el Deporte y la Salud, Facultad de Ciencias Médicas, Universidad de Santiago de Chile, USACH, Santiago, Chile; 2 Endocrine Section-Applied Physiology Laboratory, University of North Carolina at Chapel Hill, Chapel Hill, North Carolina, United States of America; 3 Centro de Estudios para la Medición de la Actividad Física «CEMA». Escuela de Medicina y Ciencias de la Salud, Universidad del Rosario, Bogotá D.C, Colombia; Fondazione Toscana Gabriele Monasterio, ITALY

## Abstract

High levels of gamma glutamyltransferase (gamma-GT) and alanine aminotransferase (ALT), as well as fatty liver index (FLI) has been associated with higher cardiovascular disease risk factors in adults. The aim of this study was to examine the relationship between gamma-GT, ALT, and fatty liver index FLI levels across a gradient number of ideal cardiovascular health metrics in a representative sample of adults from the Chilean National Health Survey 2009–2010. Data from 1,023 men and 1,449 women (≥ 15 years) from the Chilean Health Survey 2009–2010 were analyzed. Ideal cardiovascular health was defined as meeting ideal levels of the following components: four behaviours (smoking, body mass index, physical activity and diet adherence) and three factors (total cholesterol, blood pressure and fasting glucose). Adults were grouped into three categories according to their number of ideal cardiovascular health metrics: ideal (5–7 metrics), intermediate (3–4 metrics), and poor (0–2 metrics). Blood levels of gamma-GT and ALT were measured and the FLI was calculated. A higher number of ideal cardiovascular health index metric was associated with lower gamma-GT, ALT and FLI (p from trend analysis <0.001). Also, adults meeting at least 3–4 metrics were predicted less likely to have prevalence of abnormal levels of gamma-GT and FLI (p<0.001) compared to adults who met only 0–2 metrics. These findings reinforce the usefulness of the ideal cardiovascular health metrics proposed by the American Heart Association as a tool to identify target subjects and promote cardiovascular health in South-American adults.

## Introduction

Nonalcoholic fatty liver disease (NAFLD) is a common liver disease seen in clinical practice, which has reached epidemic proportions and, currently, is the most common of chronic liver disease in Western Countries [1]. NAFLD is characterized by the increased accumulation of lipid in the liver, which can stemmed from multiple factors. The most important pathological mechanisms in hepatic steatosis involve increased visceral adipose tissue secretion of proinflammatory cytokines and adipokines and release of free fatty acids into the portal system and systemic circulation, causing dyslipidemia and systemic insulin resistance [2]. The role of NAFLD as a potential independent non-communicable risk factor has recently gained considerable attention [3].

High levels of gamma glutamyltransferase (gamma-GT) and alanine aminotransferase (ALT), as well as the fatty liver index (FLI) have been associated with high CVD risk factors and their clustering in adults [4]. In a previous epidemiological prospective cohort surveys [5], elevated ALT activity was associated with all-cause/cause specific (including CVD and liver disease) mortality in long-term follow-up duration research. Furthermore, ALT may also be a good indicator of overall health [6], particularly in the context of obesity, the metabolic syndrome, and presence of cardiovascular disease [7, 8], as many patients affected by these conditions also are at risk of having and NAFLD [9, 10].

In response to the increasing burden of non-communicable diseases, the American Heart Association’s (AHA’s) 2020 Strategic Impact Goals were established for the purpose of defining the concept of “ideal cardiovascular health (CVH)” and the metrics needed for monitoring it across populations [11]. Seven metrics for CVH for adults (age ≥20 years) were identified; comprised of 4 health behaviors (current smoking, body mass index, physical activity, and healthy diet score), and 3 health physiologic factors (total cholesterol, blood pressure [BP], and fasting plasma glucose).

Nevertheless, it is not clear whether the relationship between the early liver biomarkers and CVD process is a consequence of shared established risk factors (e.g., obesity, dyslipidemia, physical inactivity, and dysglycemia) or whether it is determined by specific circulating hepatic factors (e.g., liver enzymes) or increase fatty liver estimated by a validated surrogate marker (i.e., the FLI) that originate either from liver tissue and is known to participate in the development and progression of NAFLD [12, 13]. However, these associations have been inconsistent and controversial as some epidemiology studies have failed to observe a significant association between liver biomarkers and ideal CVH behaviors/factors.

Poor diet, obesity and physical inactivity are leading CVD risk factors among Chilean adults, raising concerns about whether an increased risk of these conditions also is manifested in cardiometabolic health [14, 15]. Additionally, the prevalence of hepatic steatosis as defined by elevated FLI has not been examined in Chilean population. To address these issues we analyzed data from Chilean National Health Survey 2009–2010 examining the relationship between gamma-GT, ALT and FLI levels with and the number of ideal CVH behaviors/factors in a representative sample of adults in Chile.

## Methods

### Participants and study design

The Chilean National Health Survey of 2009–10 is a representative household survey with a stratified multistage probability sample of 5,416 participants over 15 years old from the 15 Chilean regions, both urban and rural. The sample size was calculated with 20% relative sampling error for an estimation of national representation over 4%. Only one participant was randomly selected per household, pregnant women were excluded. The response rate was 85% with no replacements. Detailed information about the survey has been described elsewhere [16]. For this study we only considered a random subsample of 2,472 adults that had completed all interest variables (i.e., ideal CVH metrics). The study was approved by the institutional review board for the use of human subject research in addition to the Pontificia Universidad Católica de Chile Board (Code N° 09–113). The protocol was in accordance with the latest revision of the Declaration of Helsinki (as revised in Hong Kong in 1989 and in Edinburgh, Scotland, in 2000) and current Chilean laws governing clinical research on human subjects (Law 20.120, at 22.09.2006, of the Ministry of Health). All subjects provided informed consent after the experimental procedures were explained. Further details can be obtained from the website (http://web.minsal.cl/estudios-y-encuestas-de-salud/).

### Measurements

Standardized protocols were used and all investigators (nurses and research technicians) underwent joint training sessions prior to the survey.

#### Anthropometrics assessment

Height was measured to the nearest 0.1 cm using a portable stadiometer and weight was measured to the nearest 0.1 kg using a digital scale (Tanita HD-313®) with participants removing their shoes and wearing light clothing at the subjects home. Waist circumference (WC) was measured in the mid-axillary line at the midpoint between the costal margin and the iliac crest. BMI was calculated as [weight/height^2^] and classified using the World Health Organization (WHO) criteria [17].

#### Resting blood pressure

Systolic blood pressure and diastolic blood pressure were measured three times within a 5-min interval using a validated HEM-742® sphygmomanometer (Bannockburn, Illinois, USA) at the subject’s home.

#### Biochemical determinations

Blood samples were obtained from each subject early in the morning, following a 10-hour overnight fast by venipuncture from the antecubital vein. Samples were centrifuged at 3000 *g* for 5 min at 4°C. Separated the serum was aspirated and aliquoted into cryovials. Finally, all samples were stored at −80°C for analysis of serum lipids and liver enzymes at a later time. Glucose, Hexokinase method (Siemens Advia 1600/1800 Erlangen; Total cholesterol (TC) CHOD-POD enzymatic method (Siemens Advia 1600/1800); Triglycerides, enzyme glycerol phosphate oxidase method (GPO) (Siemens Advia 1600/1800 Erlangen, Germany); gamma-GT, and ALT enzymatic method (Roche/Hitachi Modular P800 Tokyo, Japan). Abnormal gamma-GT (> 42 U/L for men and > 24 U/L for women <19 years old and > 50 U/L for adults in both sexes) and ALT (> 30 U/L for men and > 20 U/L for women <19 years old and > 55 U/L and <30 U/L for adults men and women, respectively) values were pre-defined by the clinical laboratory performing the analysis, Pontificia Universidad Católica de Chile [16].

FLI is a continuous score based on four variables that are simple and inexpensive to collect: BMI, WC, serum triglycerides and gamma-GT [12] using the following Eq 1:

$$FLI=\frac{e^{\left(0.953\;x\;ln\left(TG\right)\;+\;0.139\;x\;BMI\;+\;0.718\;x\;ln\left(GGT\right)\;+\;0.053\;x\;WC\;-\;15.745\right)}}{\left(1+\;e^{\left(0.953\;x\;ln\left(TG\right)\;+\;0.139\;x\;BMI\;+\;0.718\;x\;ln\left(GGT\right)\;+\;0.053\;x\;WC\;-\;15.745\right)}\right)\;x\;100}$$ (1)

The FLI was originally developed in a population residing in Northern Italy to predict the presence of NAFLD [12]. Overall, the performance of FLI for the diagnosis of NAFLD has been viewed as satisfactory in cohorts of Caucasian ethnicity [18, 19]. The FLI score ranges from 0 to 100. A FLI <30 ruled “out” and a FLI ≥60 ruled “in” for indicating poor fatty liver status with a good diagnostic accuracy [12, 20].

#### Physical activity

The Global Physical Activity Questionnaire (GPAQ) (version 2) was used to measure physical activity. The physically active category was defined as ≥150 min of moderate activity per week or >20 min of intense physical activity at least three times a week [21].

#### Healthy diet score

Consumption frequency of fish, shellfish, or other seafood (≥ 1 servings per week), whole grains (daily), fruits (≥ 2 servings per day), vegetables (≥ 3 servings per day) and sodium (<15 g/d) were analyzing using a healthy diet score (4–5 scores) by a questionnaire designed ad-hoc [16]. Adults with a healthy diet score of 4 were assigned to ideal diet [11].

#### Ideal Cardiovascular health

The American Health Association (AHA) guidelines were used to construct an ideal CVH index of the seven metrics using the cut-off points for adults, with the participants given one point for the presence of each ideal metric. The behaviors defined by AHA were as follows: BMI <25 kg/m^2^, ≥600 MET min per week, not smoking (either never having smoked or quit smoking >12 months ago), and consumption of a dietary pattern that promotes ideal CVH [11]. The factors were classified as: untreated systolic BP <120 mm Hg and diastolic BP <80 mm Hg, untreated total cholesterol ≤200 mg/dL, and untreated fasting glucose <100 mg/dL.

Finally, each participant was categorized into 1 of 3 health levels based on the number of ideal CVH metrics in the ideal range; the healthiest level (favorable ideal CVH score) was defined as having between 5 to 7 metrics, the intermediate level as 3 to 4 metrics in the ideal range and the unfavorable level as having 0 to 2 ideal metrics. These cut-off points were used in prior international studies [22, 23].

#### Socio-demographics

Socio-demographic data were collected for all participants, including age, gender, education level (primary, secondary or beyond secondary), years of schooling, and monthly household income (was stratified into four categories: ≤US $247.00 [lowest], US $248.00–452.00 [medium lowest], US $453.00–1180.00 [medium highest] and > US $1180.00 [highest]) [14], alcohol intake was assessed by Alcohol Use Disorders Identification Test (AUDIT) [24], and smoking status (non-smoker, past-smoker or smoker). Past smokers were those who reported that they had smoked > 100 cigarettes during their lifetime but did not currently smoke cigarettes. The AUDIT test identifies dangerous drinking behaviors through a summary score constructed from questions on drinking behavior such as drinking frequency, quantity, or not being able to stop drinking.

### Statistical analysis

Statistical normality was tested using both statistical (Kolmogorov–Smirnov test) and graphical procedures (normal probability plots). Due to their skewed distribution, gamma-GT and ALT were log-transformed. To aid interpretation, data were back-transformed from the log scale for presentation in the results. Since no significant interaction was observed between sexes (e.g., sex and ideal CVH metrics), to increase statistical power, all the statistical analysis was performed with both sexes combined together. Means of liver enzymes were calculated for each ideal CVH metrics and compared using the Jonckheere–Terpstra test for trends. Finally, logistic regression models were employed to compare the prevalence of abnormal hepatic parameters values across individual and number of ideal CVH. All models were adjusted for age, sex, income, education level, medication use, and alcohol intake. All analyses were conducted with the use of the complex survey design routines of the SPSS Statistical software package (Software, v.22.0 SPSS Inc., Chicago, IL, USA), and a value of *P* < 0.05 was considered to be statistically significant.

## Results

Descriptive characteristics, stratified by gender are presented in Table 1. The cohort comprised 2,472 adults (mean age 46.0 years old) and 58.6% were women. The prevalence of ideal healthy diet score and total cholesterol level were similar for both men and women.

**Table 1:** Baseline demographic and clinical characteristics of subjects.

	Total (n = 2.472)	Men (n = 1.023)	Women (n = 1.449)	P for sex
Age (years)	46.0 (18.3)	45.5 (18.3)	46.4 (18.2)	0.250
Weight, kg	**71.7 (14.9)**	**77.5 (14.4)**	**67.7 (13.9)**	**<0.001**
Height, cm	**160.6 (9.6)**	**168.2 (7.4)**	**155.3 (7.0)**	**<0.001**
Body mass index, kg/m^2^	**27.8 (5.3)**	**27.4 (4.8)**	**28.1 (5.7)**	**0.001**
Waist circumference, cm	**93.1 (41.0)**	**95.5 (39.5)**	**91.4 (41.9)**	**<0.001**
Moderate physical activity, min per day	**190.7 (212.1)**	**235.1 (232.5)**	**159.3 (190.5)**	**<0.001**
Fruits and vegetables (≥ 5 servings per day). n (%)	**1574 (63.7)**	**582 (56.9)**	**992 (68.5)**	**<0.001**
Fish (≥ 1 servings per week). n (%)	292 (11.8)	116 (11.3)	176 (12.1)	0.292
Whole grains (every day). n (%)	**295 (11.9)**	**81 (7.9)**	**214 (14.8)**	**<0.001**
Sodium intake, g/day	**9.6 (2.3)**	**9.8 (2.3)**	**9.4 (2.3)**	**<0.001**
Office systolic blood pressure, mmHg	**127.7 (22.2)**	**132.8 (21.6)**	**124.2 (22.0)**	**<0.001**
Office diastolic blood pressure, mmHg	**76.3 (11.2)**	**78.9 (11.7)**	**74.3 (10.6)**	**<0.001**
Antihypertensive drug. n (%)	**325 (13.1)**	**111 (10.9)**	**214 (14.8)**	**<0.001**
Total cholesterol, mg/dL	194.7 (43.4)	195.2 (44.4)	194.2 (42.5)	0.824
Triglycerides, mg/dL	**149.9 (120.7)**	**168.3 (151.3)**	**136.9 (91.1)**	**<0.001**
Fasting glucose, mg/dL	**95.6 (29.3)**	**98.4 (30.8)**	**93.7 (28.1)**	**<0.001**
Antidiabetic drug. n (%)	154 (6.2)	63 (6.2)	91 (6.3)	0.914
Alcohol use risk. n (%)	**647 (26.2)**	**433 (42.3)**	**214 (14.8)**	**<0.001**
Education				
Up to primary (<8 years). n (%)	**781 (31.6)**	**321 (31.4)**	**460 (31.7)**	0.009
Up to secondary (<12 years). n (%)	**914 (37.0)**	**350 (34.2)**	**564 (38.9)**	
Beyond secondary (>12 years). n (%)	**704 (28.5)**	**326 (31.9)**	**378 (26.1)**	
Income				
Lowest. n (%)	**474 (19.2)**	**164 (16.0)**	**310 (31.4)**	**<0.001**
Medium lowest. n (%)	**824 (33.3)**	**324 (31.7)**	**500 (34.5)**	
Medium highest. n (%)	**794 (32.1)**	**357 (34.9)**	**437 (30.2)**	
Highest. n (%)	**294 (11.9)**	**147 (14.4)**	**147 (10.1)**	
Gamma-Glutamyltransferase, U/L	**32.9 (41.7)**	**38.9 (42.7)**	**28.8 (40.5)**	**<0.001**
High, n (%)	**353 (14.3)**	**208 (20.3)**	**145 (10.0)**	**<0.001**
Alanine Aminotransferase, U/L	**26.4 (21.2)**	**31.0 (24.0)**	**23.1 (18.4)**	**<0.001**
High, n (%)	**344 (13.9)**	**102 (10.0)**	**242 (16.7)**	**<0.001**
Fatty liver index	**46.9 (31.1)**	**51.8 (29.8)**	**43.5 (31.5)**	**<0.001**
Fatty liver, n (%)*	**962 (38.9)**	**458 (44.8)**	**504 (34.8)**	**<0.001**
Goal/Metric				
Not currently smoking, n (%)	**955 (38.6)**	**431 (42.1)**	**524 (36.2)**	**0.003**
Body mass index <25 kg/m^2^, n (%)	**1748 (70.7)**	**783 (76.5)**	**965 (66.6)**	**<0.001**
Physically active, n (%)	**1685 (68.2)**	**752 (73.5)**	**933 (64.4)**	**0.013**
Healthy Diet Score, n (%)	44 (1.8)	10 (1.0)	34 (2.3)	0.072
Total cholesterol <200 mg/dL, n (%)	1402 (56.7)	562 (54.9)	840 (58.0)	0.072
Fasting glucose <100 mg/dL, n (%)	2244 (90.8)	920 (89.9)	1324 (91.4)	0.125
Optimal blood pressure, n (%)	**1693 (68.5)**	**671 (65.6)**	**1022 (70.5)**	**<0.001**

Values are mean (SD) and number and proportions (n (%)) for categorical data.

* FLI ≥60 at risk.

Table 2 describes liver enzyme levels in Chilean adults with ideal and non-ideal CVH behaviors and factors individually. Gamma-GT levels were higher in subjects with non-ideal BMI (P < 0.001) and non-ideal total cholesterol (P < 0.001), glucose (P = 0.001) and blood pressure (P < 0.001) factors, compared with those participants meeting the ideal CVH criteria. ALT levels were higher in adults with non-ideal smoking and BMI health behaviors (P < 0.001) and non-ideal total cholesterol (P < 0.001) and glucose (P < 0.001) health factors than in those participants meeting these ideal CVH behaviors and factors criteria. Finally, FLI was higher in adults with non-ideal BMI, physical activity, total cholesterol, glucose and blood pressure (all P < 0.001) compared with those adults meeting these ideal CVH criteria.

**Table 2:** Mean differences of liver enzymes levels according to ideal cardiovascular health metrics.

	Gamma-Glutamyltransferase			Alanine Aminotransferase			Fatty liver index (0–100)		
	Ideal	Non-Ideal	P	Ideal	Non-Ideal	P	Ideal	Non-Ideal	P
Health behaviors									
Smoking	33.0 (1.4)	32.9 (1.1)	0.990	**25.9 (0.8)**	**26.7 (0.6)**	**<0.001**	48.5 (1.0)	45.7 (0.8)	0.509
BMI	**29.4 (0.9)**	**40.6 (1.5)**	**<0.001**	**23.1 (0.5)**	**33.9 (0.8)**	**<0.001**	**33.4 (0.5)**	**79.1 (0.8)**	**<0.001**
Physical activity	32.7 (1.0)	33.5 (1.5)	0.649	26.3 (0.5)	26.3 (0.8)	0.989	**46.0 (0.7)**	**49.0 (1.0)**	**0.024**
Diet	38.1 (6.3)	32.9 (0.8)	0.406	28.3 (3.4)	26.3 (0.4)	0.557	41.4 (4.4)	47.1 (0.6)	0.208
Health factors									
Total cholesterol	**28.9 (1.1)**	**38.3 (1.3)**	**<0.001**	**23.1 (0.6)**	**30.5 (0.7)**	**<0.001**	**40.9 (0.8)**	**44.1 (0.9)**	**<0.001**
Glucose	**32.1 (0.9)**	**41.9 (2.8)**	**0.001**	**25.5 (33.1)**	**33.1 (1.4)**	**<0.001**	**45.6 (0.6)**	**62.2 (2.0)**	**<0.001**
Blood pressure	**31.7 (1.1)**	**35.5 (1.7)**	**<0.001**	25.9 (0.6)	27.4 (0.9)	0.203	**43.5 (0.7)**	**54.1 (1.2)**	**<0.001**

Data are means ± SE. Adjusted by age, sex, income, education level, medication use, and alcohol use

**Fig 1:**
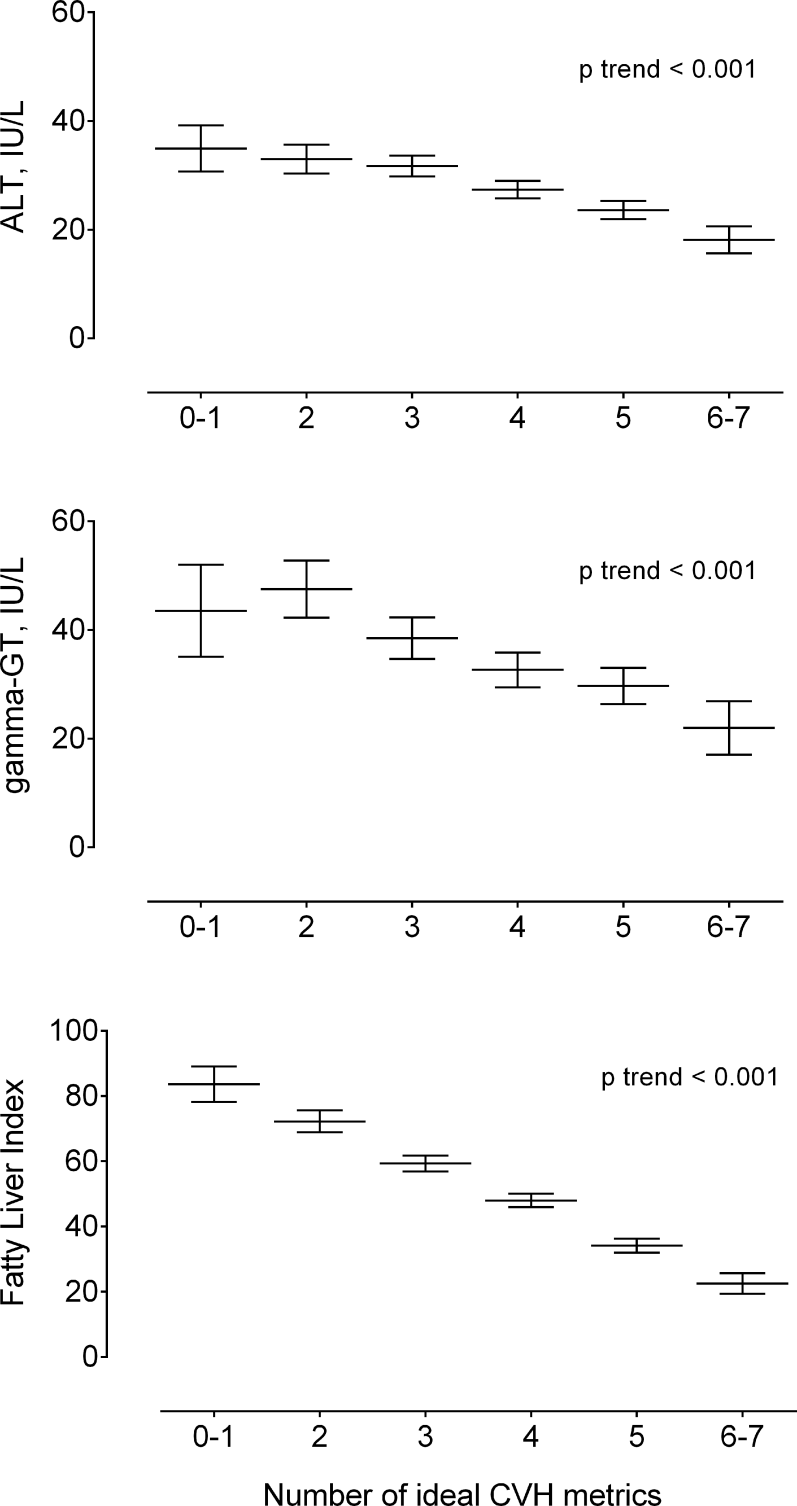
Association between gamma-GT, ALT, and FLI with the number of components of the ideal CVH in Chilean adults.

Fig 1 describes the characteristics of the study population in all ideal CVH metrics for each liver enzymes (gamma-GT, ALT) and FLI. The figure shows a significant trend across metrics for all variables (p < 0.001).

Table 3 shows odds ratio (OR) for prevalence of abnormal liver enzymes levels according to individual and number of ideal CVH metrics. Adjusted analysis suggests that ideal fasting glucose was less likely to have prevalence of abnormal levels of gamma-GT (OR = 0.65 95% CI, 0.45 to 0.94), ALT (OR = 0.59 95% CI, 0.46 to 0.76), and FLI (OR = 0.44 95% CI, 0.29 to 0.65) than adults with non-ideal glucose levels. Also, adults with ideal BMI were less likely to have a prevalence of abnormal levels of gamma-GT (OR = 0.49 95% CI, 0.38 to 0.63) and ALT (OR = 0.31 95% CI, 0.25 to 0.41), ideal total cholesterol for ALT (OR = 0.59 95% CI, 0.46 to 0.76) and FLI (OR = 0.35 95% CI, 0.28 to 0.45) and physically active adults for FLI (OR = 0.83 95% CI, 0.65 to 0.92). Finally, accordly the number of ideal CVH metrics, intermediate (3–4 metrics) profile predicts less likely to have prevalence of abnormal levels of gamma-GT (OR = 0.57 95% CI, 0.42 to 0.77) and FLI (OR = 0.23 95% CI, 0.18 to 0.31) and ideal profile (5–7 metrics) predicts less likely to have prevalence of abnormal levels of gamma-GT (OR = 0.27 95% CI, 0.18 to 0.40), ALT (OR = 0.28 95% CI, 0.19 to 0.43), and FLI (OR = 0.05 95% CI, 0.03 to 0.07).

**Table 3:** Odds ratio for risk of abnormal liver enzymes levels according to individual and number of ideal cardiovascular health metrics.

	Gamma-Glutamyltransferase			Alanine Aminotransferase					Fatty liver index ≥ 60		
	OR	(95% CI)	P	OR		(95% CI)	P		OR	(95% CI)	P
Health metrics											
Smoking†	1.14	1.01 to 1.80	0.326	0.80		0.62 to 1.04	0.099		1.11	0.87 to 1.41	0.379
Body mass index†	**0.49**	**0.38 to 0.63**	**<0.001**	**0.31**		**0.25 to 0.41**	**<0.001**			#	
Physically active†	0.89	0.69 to 1.16	0.419	1.21		0.92 to 1.58	0.155		**0.83**	**0.65 to 0.92**	**0.037**
Healthy Diet Score†	1.26	0.51 to 3.10	0.610	1.29		0.56 to 2.97	0.535		0.87	0.34 to 2.05	0.748
Total cholesterol†	0.46	0.36 to 0.58	0.456	**0.59**		**0.46 to 0.76**	**<0.001**		**0.35**	**0.28 to 0.45**	**<0.001**
Fasting glucose†	**0.65**	**0.45 to 0.94**	**0.021**	**0.51**		**0.35 to 0.76**	**0.001**		**0.44**	**0.29 to 0.65**	**<0.001**
Blood pressure†	0.78	0.59 to 1.04	0.097	0.88		0.65 to 1.21	0.455		**0.71**	**0.54 to 0.94**	**0.017**
No. Ideal Cardiovascular Health metrics											
Poor (0–2)*		ref			ref					ref	
Intermediate (3–4)*	**0.57**	**0.42 to 0.77**	**<0.001**	0.72		0.52 to 1.01	0.057		**0.23**	**0.18 to 0.31**	**<0.001**
Ideal (5–7)*	**0.27**	**0.18 to 0.40**	**<0.001**	**0.28**		**0.19 to 0.43**	**<0.001**		**0.05**	**0.03 to 0.07**	**<0.001**

# Was not included due to possible collinearity.

* Adjusted by age, sex, income, education level, medication use, and alcohol use

† Adjusted by previous variables and the whole of individual metrics.

## Discussion

The findings of the current study show a negative relationship between liver enzymes levels and the number of ideal CVH in a representative sample of adults from Chile. Previous observational studies have examined the associations of liver enzyme levels, as surrogate indicators of liver fibrosis and NAFLD, and with cardiometabolic risk factors in adult population [25, 26]. However, as far as we are aware, this is the first study examining the influence of the ideal CVH metrics on liver enzymes, specifically in a South American population.

Biologically, elevated fasting glucose will result from hepatic insulin resistance, whereas increased free fatty acids concentrations are the expression of peripheral insulin resistance [27]. Additionally, a fatty liver has been proposed as the hepatic manifestation of the metabolic syndrome which includes high blood pressure, hyperglycemia, central adiposity and dyslipidemia [27]. More recently, several studies reported the high gamma-GT, ALT and FLI values have been associated with elevated levels of total and LDL cholesterol, triglycerides [28], insulin levels [29], early carotid plaques [4] and endothelial dysfunction [30].

One of the most interesting findings of the current study is the significant association of the ideal CVH metrics (behaviors/risk factors) with liver enzymes in a Chilean adult population. In adults, the influence of the ideal CVH behaviors index on liver biomarker levels has not been previously examined, which hampers comparisons with other studies. In youth population however, Labayen et al. [31] showed a positive association between a higher number of ideal CVH metrics behaviors/factors with lower gamma-GT and ALT in 637 European adolescents. Similarly, Labayen et al. [32] observed a lower aspartate aminotransferase/ALT ratio was also associated with higher cardiometabolic risk factors and their clustering in 1,084 male and female European adolescents. Regarding the FLI (*an algorithm for predicting fatty liver*), few previous studies have reported on the relationship between FLI and cardiometabolic risk factors and their clustering in adults. Kozakova et al. [4] found in subjects with FLI ≥ 60 had a higher carotid plaques and a lower plasma adiponectin level, as compared to those with FLI <60; they also showed an inverse relationship between plasma adiponectin and FLI. Similarly, Jäger et al. [33] found a strong positive association between the FLI, as a surrogate measure for fatty liver, and the incident of Type 2 Diabetes independent of potential confounding such as socioeconomic and lifestyle risk factors in 1,922 from The European Prospective Investigation into Cancer and Nutrition (EPIC)-Potsdam study.

Nevertheless, several studies reported significant relationships of some of the behavior factors considered in the ideal CVH behavior index with liver surrogate markers. Thus, our results confirm previous findings, which are mainly based on studies in youth and non-Latin adults populations and on cross-sectional data. That is, for example, a study of 15,586 Japanese workers showed a positive correlation between higher levels of gamma-GT in smoker than those in non-smoker adults between 40 and 54 years of age [34]. This is further supported by Nakanishi et al. [35] and Kim et al. [36], who showed raised gamma-GT and ALT levels in smoker compared to non-smoker peers. However, cigarette smoking impacts all phases of carotid artery intimal hyperplasia from endothelial dysfunction [4, 37], and one of the mechanisms that may participate in NAFLD in smokers is the effect of nicotine on liver injury (confirmed in animal models) [38]. Additionally, circulating cigarette smoke constituents seem to play an important role in the underlying molecular mechanisms for some aspects of muscle damage, such as reduced oxygen delivery and impair mitochondrial function [39].

On the other hand, it is known that vigorous to regular physical activity is associated with a healthier metabolic profile [40], insulin sensitivity [41] as well as lower total and central adiposity [42] in adults. Increases in ideal CVH are directly associated with muscular fitness [43] and healthier levels of cardiorespiratory fitness in adolescents [44]. In fact, several epidemiological cross-sectional [45] and retrospective studies [36] already revealed a significant association between higher levels of physical activity and a lower prevalence of NAFLD. Recent evidence also suggests that physical activity may play a key role on hepatic fat content by directly altering hepatic β-oxidation and/or lipogenesis [46]. The activation of AMP-kinase increases ATP production through fatty acid oxidation and glucose transport, and AMP-activated protein kinase is activated by depletion of ATP such as in the case of exercise training. Our results shown that FLI levels is related positively with physical activity levels. Along this line, it is biologically plausible then that higher physical activity levels are associated with fatty liver enzyme levels at this age [47].

The AHA includes BMI as one of four health behaviors to track for the 2020 Strategic Impact Goals. Consistent with our findings, previous studies observed higher ALT and gamma-GT levels in weight excess than in healthy weight adults [48]. Indeed, the strong association between BMI and liver enzyme levels is consistent across several different studies [7, 29]. There is a solid body of evidence for the association of nutritional profile with CVD, especially the influence of healthy dietary patterns on liver surrogate markers has been previously reported in adults [49]. In our study, we did not detect a significant effect of healthy dietary behaviors on liver enzymes. Thus, adults meeting with the ideal CVH diet behavior, as defined by the AHA, did not show lower ALT and gamma-GT levels, and there was not a trend to higher FLI. Previously, it has been shown in adults, higher adherence to a Mediterranean diet style was associated with lower levels of ALT and gamma-GT [50]. However, evidence suggests that the strong involvement of dietary composition in the pathogenesis of NAFLD may be responsible for the controversial findings on the role of diet composition, in terms of macro- or micronutrients, in the pathogenesis of this disease [51].

Our logistic regression analyses showed that BMI and fasting glucose predicts abnormal liver enzymes levels (gamma-GT, ALT levels, and FLI). These associations may be the consequence of a link between excess adipose tissue and hepatic insulin resistance mediated by increased free fatty acids to the liver (fat overflow) from adipocytes, leading to higher hepatic lipogenesis and trygliceride-rich lipoprotein secretion [31]. Moreover, hypertriglyceridemia is more commonly associated with raised hepato-biliary enzymes, and particularly gamma-GT [25], an association which appears to be independently mediated by insulin resistance and fatty liver status.

Collectively, these results further extend the evidence of the importance of meeting at least four ideal cardiovascular health components of the seven metrics proposed by the AHA for cardiovascular health in adults [11]. In this context, surveillance of ideal CVH is particularly important in Latin America, where most of the burden is due to unhealthy behaviors and related to non-communicable chronic diseases [52].

### Limitations

This study has some limitations. First, the cross-sectional design does not allow us to make cause–effect inferences between liver enzymes and ideal CVH components. Second, is that we are not able to exclude that liver enzyme concentrations could have been affected by viral hepatitis or drug -ingestion. However, participants were examined by medical doctors and all of them had a negative history of liver disease. Another limitation was that diet and physical activity levels were measured by a self-administered questionnaire, so some of the questions may have been misinterpreted deliberately or unintentionally by some participants. However, the assessment of a relatively large sample of subjects not selected for NAFLD or obesity should be considered as a counter-balancing strength of this study. Another limitation is the lack of a caloric measure of total energy fat and sugars intake, which was unable to be obtained from the questionnaire used. Finally, although we can not confirm that the enzymes analyzed were completely of hepatic origin, we can state that most transmaminase is produced by the liver, so this slight limitation does not affect the results. Despite these limitations, the study also has various strong points. In particular, the results present for the first time, the relationship between ALT, gamma-GT and FLI levels with a higher number of ideal CVH behaviors/factors on a large representative sample of Chilean adults.

## Conclusions

In summary, we show that three liver enzymes are associated with ideal CVH behaviors/factors in a representative sample of adults from Chile. These findings reinforce the usefulness of the CVH proposed by the AHA as a tool to identify target subjects and promote CVH in adults and extend these observations to the liver manifestation of premature onset of metabolic syndrome, cardiovascular disease, Type 2 Diabetes, and NAFLD. Longitudinal studies are required to establish whether ideal CVH behaviors/factors represents independent predictors of NAFLD, and whether may help to identify patients at risk of progressive disease.
